# Predictors of Hand Grip Strength in Adults Without Sarcopenia: Data From the NHANES, 2013–2014

**DOI:** 10.1016/j.cdnut.2024.102149

**Published:** 2024-04-03

**Authors:** Mansour M Alotaibi

**Affiliations:** 1 Department of Rehabilitation, College of Applied Medical Sciences, Northern Border University, Arar, Saudi Arabia; 2 Center for Health Research, Northern Border Universit, Arar, Saudi Arabia

**Keywords:** body composition, adiposity, muscle strength, bone mineral density, lean mass

## Abstract

**Background:**

Grip strength measurement is used to estimate muscle strength and predict health status; yet, an accurate examination of grip strength predictors from body composition variable is lacking.

**Objectives:**

This study aimed to examine the association of grip strength with lumbar bone mineral density (BMD) and total lean mass in adults without sarcopenia.

**Methods:**

Adults without sarcopenia (*N* = 3100) were included from the NHANES, 2013–2014, in this cross-sectional study. Body mass (kg), body height (cm), body mass index (kg/m^2^), grip strength (kg), total percent fat (%), lumbar BMD (g/cm^2^), and total lean mass excluding bone mineral content (BMC, kg) were obtained and tested as predictors of grip strength.

**Results:**

The regression analysis yielded a significant model [*F*(2,343732) = 71,284.2; *R*^2^ = 0.713; *P* < 0.001], with all predictors explaining ∼71.3% of the variance in grip strength. Age [β: −0.043; 95% confidence interval (CI): −0.040, −0.036], sex (β: −0.296; 95% CI: −6.431, −6.270), total percent fat (β: −0.245; 95% CI: −0.315, −0.308), lumbar BMD (β: 0.037; 95% CI: 2.529, 2.806), and total lean mass (β: 0.482; 95% CI: 0.001, 0.001) were all significant predictors of grip strength.

**Conclusions:**

The predictive value of the BMD and total lean mass can serve as a useful measure in predicting grip strength and overall health status in adults without sarcopenia.

## Introduction

Measurements of grip strength are widely used, more commonly in older adults, to estimate overall strength and predict future health status [1]. Studies have reported that measuring grip strength is reliable and valid in estimating overall strength in healthy adults [2,3]. More specifically, grip strength measured by a dynamometer, reflects the function of the upper extremities of which the hands are part [4]. Research by Giray and Akyϋz [5] found a moderate but significant correlation between (*r* = −0.32) between grip strength and self-reported upper extremity function, measured by the Disabilities of the Arm, Shoulder, and Hand questionnaire in women postmastectomy lymphedema [5]. Although grip strength is not directly involved in performing functional activities such as raising from a chair, weak grip strength is associated with physical limitations and poor health status in older adults [6]. Furthermore, grip strength is a predictor of multiple future outcome measures and, more importantly, premature mortality [1,7]. For example, multiple hazard ratios (HRs) categorized by 5-kg decrease in grip strength were all associated with all-cause mortality (lowest HR: 1.15; highest HR: 1.23; all *P* < 0.05) in middle-aged and older adults in the United Kingdom [8]. Weak grip strength is also associated with cardiovascular disease, all respiratory disease, chronic obstructive lung disease, and different types of cancer [1,9]. Collectively, grip strength is an important proxy marker for the overall health status, function, and preventing premature mortality.

Demographic factors, specifically age and sex, explain a proportion of the variation in grip strength [10,11]. Despite the heterogeneity in measuring grip strength, grip strength usually increases in early adulthood onward until it reaches a peak through mid-life (∼30–40 y of age) and then subsequently declines with age [10,11]. Demographic variables, such as age and sex, can moderate grip strength readings in young adults through older adults [12]. Several grip strength normative data for adults showed differences in this measure by age and sex in the United States [13,14], European [10,15,16], and Asian [17] populations. Adiposity, determined by BMI, percent fat, and waist circumference, is linked to multiple negative health outcomes, including a decline in function and premature mortality [18]. Increased adipose tissue could further lead to the redistribution of fat creating fatty infiltration in the skeletal muscles, which deteriorates skeletal muscle quality and function and increase risk of developing sarcopenia [19]. To these ends, controlling for confounding factors such as age, sex, and adiposity is important when examining grip strength.

Other body composition factors such as lean mass and bone mineral density (BMD) may play a role in the measurement of grip strength. Lean mass can be evaluated using different validated imaging techniques, including dual-energy X-ray absorptiometry (DXA), or bioimpedance analysis devices [20]. DXA absorptiometry also measures BMD at different sites of the skeletal system, which guides clinicians in identifying issues with BMD and diagnosing osteoporosis [21]. The cumulative increase in age-related inflammatory factors and the fat redistribution could impact the quality of skeletal muscle and bones [22], which subsequently reduces the overall muscle function, as reflected by grip strength [23]. Researchers found that an increase of 1 unit in lumbar BMD was associated with a 93% reduction in risk of developing sarcopenia [24], which provides promising evidence to prevent sarcopenia though improving BMD. Although the association of grip strength with BMD and total lean mass is well established [25,26], little is known about the relationship among these variables while controlling for age, sex, and percent fat. Identifying these relationships will provide information for clinicians and researchers on how to improve grip strength through implementation of strategies that increase BMD and lean mass, given the value of grip strength in predicting function and overall health status.

This study aimed to determine the association of lumbar BMD and total lean mass with grip strength in United States adults without sarcopenia, controlling for age, sex, and total percent fat using data from the NHANES, 2013–2014. The hypothesis was that lumbar BMD and total lean mass will significantly predict grip strength, after controlling for age, sex, and total percent fat.

## Methods

### NHANES overview

The NHANES is a nationally representative survey designed to assess the health and nutritional status of the civilian, noninstitutionalized population. NHANES program is conducted by the National Center for Health Statistics (NCHS), which is a part of the Centers for Disease Control and Prevention (CDC) in the United States [27]. Briefly, NHANES collects data on a wide range of health-related topics, such as chronic diseases, infectious diseases, environmental exposures, and health behaviors, to help researchers and policymakers monitor changes in health trends over time. Additionally, NHANES provides data on demographics, physical examination, laboratory tests, dietary intake, nutritional biomarkers, and nutritional deficiencies. Collectively, collecting this information helps policymakers and researchers make informed decisions, develop new health care policies, and allocate resources effectively.

### Participants and study design

Adults (men and women) aged 18–59 y were included in this cross-sectional study. Participants were excluded if they were diagnosed with sarcopenia. Participants were recruited using a multistage probability sampling design to select representatives of the civilian noninstitutionalized United States population. Sarcopenia diagnosis was based on the European Working Group on sarcopenia in Older People (EWGSOP2) definition of sarcopenia, which requires adults to have low muscle strength determined by grip strength (men, <27 kg; women <16 kg) and confirmed by low muscle quantity or quality determined by appendicular skeletal mass (men, <20 kg; women, <15 kg) [28]. This study used a retrospective cross-sectional design.

### Outcome measurements

#### Demographics and body measures

Demographic variables including age (y), sex, race, body mass (kg), and body height (cm) were collected at the Mobile Examination Center following a standard methodology described in detail in the NHANES procedures manual [29]. BMI was calculated as body mass (kg) divided by squared body height (m^2^).

#### Grip strength

The handgrip strength procedures were explained in detail elsewhere [30]. In brief, a Takei Digital Grip Strength Dynamometer (Model T.K.K.5401; Takei Medical) was used to assess grip strength (kg). Participants performed 3 trials with 60 s of rest allowed between trials. Participants alternated between hands to further increase rest time between trials. The best of the 3 trials was selected as the test score and the mean grip strength score of the 2 hands was selected as the variable. Finally, grip strength is a valid and reliable measurement of muscle strength [31].

#### Body composition

Whole-body DXA scans were administered to all participants. Adiposity (total percent fat, %), total lean mass excluding bone mineral content (kg), and lumbar BMD (g/cm^2^) were derived from DXA measures. Lumbar BMD was chosen because low lumbar BMD is a serious health concern, especially in women, and has been used frequently to track the effects of exercise interventions on BMD [32].Whole-body scans were done using Hologic Discovery Model A densitometer and postprocessed by Apex software 4.0. Radiation exposure from DXA was maintained at <20 μSv. DXA is the most widely accepted method of measuring body composition [33,34], including BMD [21].

### Statistical analyses

The Statistical Package for the Social Sciences software v29.0 (SPSS; IBM) guided all statistical analyses. Descriptive statistics included means ± SD for continuous variables (age in years, body mass, body height, BMI, grip strength total percent fat, total lean, lumbar BMD, and grip strength). Skewness and kurtosis analysis determined if there was a violation of the normality assumption. For all analysis, the full-sample 2-y interview weight was used for descriptive statistics (means and SDs), and masked variance pseudostratum was used for the inferential statistics (the independent *t* tests and regressions). In addition, there were no significant differences in the original analysis and the weighted analysis. Thus, the weighted analysis was used to drive estimates about the United States population. Independent *t* tests were used to compare continuous variables between men and women. In addition, a multivariate hierarchical linear regression model was used to determine the association between grip demographic and body composition variables with grip strength as follows: outcome was the mean grip strength score of the 2 hands (kg)—block 1 included age and sex, block 2 included total percent fat, and block 3 included lumbar BMD total lean mass. Linearity was assessed using scatterplots between grip strength and each continuous predictor, and homoscedasticity was assessed using scatterplots between the standardized residual values and the standardized predicted values. Finally, multicollinearity was assessed using the variance inflation factor, and a value of ≥5 determined a violation of the multicollinearity assumption [35]. An α level of 0.05 was the significance criterion for all statistical tests and model building.

The regression model in this study was further cross-validated by choosing a random sample of 75% of the sample (i.e., training data) to examine the model’s stability against losing the ability to predict the outcome. The adjusted *R*^2^ and the significant predictors were compared between the original and validated regression models by ensuring <2% of the adjusted *R*^2^ and no change in the significant predictors. Then, the standard error of the residual means was compared between the original and validation models by creating an equivalent standard error of estimate for the residuals in the remaining 25% of the data (i.e., the validation data) to examine the difference between the created standard error from the regression model and the equivalent standard error of the estimate to ensure the model’s validity.

## Results

### Description of participants

**FIGURE 1:**
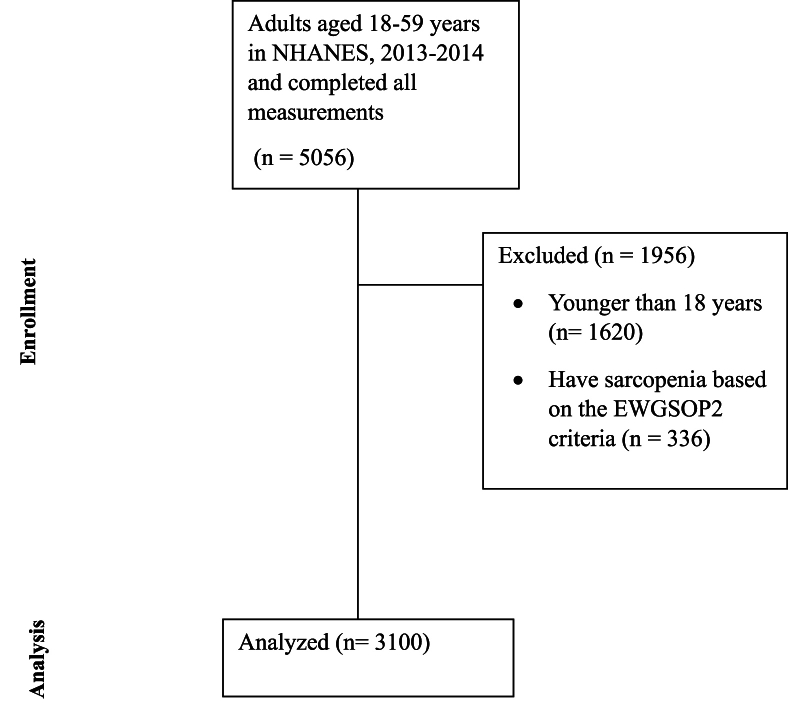
Recruitment flowchart for participants. EWGSOP2, European Working Group on Sarcopenia in Older People.

A total of 5056 completed all the measurements of interest were included from the NHANES, 2013–2014 data set. Of those, 3100 participants were included in this study (women: 49.2%; mean age, 28.6 ± 15.6 y). Participants who were younger than 18 y were excluded (*n* = 1620) and those who met the sarcopenia definition determined above were also excluded from the analysis (*n* = 336). Figure 1 summarizes the recruitment flow chart of participants. Across the sample, participants were classified as slightly overweight based on their BMI. Overall, men had significantly greater body mass, body height, BMI, grip strength, lumbar BMD, and total lean mass than women (all *P* < 0.001). Notably, men were taller and had more total lean mass than women, with a large effect size (Cohen *d* ≥ 1.61). In contrast, women had significantly greater total percent fat than men (*P* < 0.001). Table 1 compares men and women concerning demographic and clinical outcomes.

**TABLE 1:** Demographic and clinical outcomes characteristics of participants

Characteristic	Men (weighted sample) (*n* = 50,085,479)	Women (weighted sample) (*n* = 48,258,349)	*t* (1,343736), *P*	Cohen *d*	Total (weighted sample) (*N* = 130,907,005)
Age (y)	37.0 ± 12.5	37.4 ± 12.1	−9.1, <0.001	−0.03	38.0 ± 12.2
Body mass (kg)	85.6 ± 20.2	75.3 ± 20.9	145.9, <0.001	0.50	82.0 ± 21.3
Body height (cm)	174.7 ± 7.2	161.6 ± 6.7	551.0, <0.001	1.88	169.4 ± 9.6
BMI (kg/m^2^)	28.0 ± 6.1	28.8 ± 7.5	−36.0, <0.001	−0.12	28.5 ± 6.7
Grip strength (kg)	46.2 ± 8.2	29.7 ± 5.2	704.2, <0.001	2.40	38.7 ± 10.9
Lumbar BMD (g/cm^2^)	1.0 ± 0.2	1.0 ± 0.1	−11.4, <0.001	−0.04	1.0 ± 0.1
Total lean mass excluding BMC (kg)	59.5 ± 10.8	43.4 ± 9.1	472.9, <0.001	1.61	52.4 ± 12.9
Total percent fat (%)	26.9 ± 6.0	38.6 ± 6.2	−567.2, <0.001	−1.94	32.8 ± 8.3

Descriptive statistics were weighted by sample 2-y interview weights. Inferential statistics were weighted by the masked variance pseudostratum. Values are mean ± SD.

Abbreviations: BMD, bone mineral density; BMC, bone mineral content.

### Predictors of grip strength

There was no significant violation of normality and linearity assumptions based on the results of skewness, kurtosis, and scatterplots between the variables of interest. All variance inflation factor values of predictors were <5, ensuring no multicollinearity between the predictors. In addition, there was no violation of the homoscedasticity assumption, documented by the scatterplots between the standardized residual values and the standardized predicted values. The variables in the first block of the hierarchal regression predicted grip strength [*F*(2,343735) = 2,50,676.9; adjusted *R*^2^ = 0.593; *P* < 0.001]. Both age and sex were significant predictors of grip strength (all *P* < 0.001) (Table 2, first block). The addition of total percent fat in the second block slightly but significantly increased the model’s ability to predict grip strength [*F*(2,343734) = 0.000; adjusted *R*^2^ = 0.593; *P* < 0.001]. In the second block, age, sex, and total percent mass significantly predicted grip strength (all *P* < 0.001) (Table 2, second block). In the third block of the hierarchical regression, the addition of total lumbar BMD and total lean mass further increased the model’s prediction of grip strength [*F*(2,343732) = 71,284.2; adjusted *R*^2^ = 0.713; *P* < 0.001]. All predictors in the third block significantly predicted grip strength (all *P* < 0.001) (Table 2, third block). Finally, the final model documented that the predictors (age, sex, total percent fat, lean mass, and lumbar BMD) explained ∼71.3% of the variance in grip strength.

**TABLE 2:** Predictors of grip strength from hierarchical multiple linear regression analyses

Predictors	Standardized β-coefficient (entire sample)	Standardized β-coefficient (75% of the sample)	*P* (95% CI) (Entire sample)	*P* (95% CI) (75% of the sample)
First block^1^				
Age (y)	−0.051	−0.049	<0.001 (−0.047, −0.043)	<0.001 (−0.046, −0.042)
Sex	−0.768	−0.773	<0.001 (−16.534, −16.442)	<0.001 (−16.834, −16.728)
Second block^2^				
Age (y)	−0.050	−0.048	<0.001 (−0.046, −0.042)	<0.001 (−0.044, −0.040)
Sex	−0.763	−0.767	<0.001 (−16.454, −16.442)	<0.001 (−16.723, −16.574)
Total percent fat (%)	−0.007	−0.009	<0.001 (−0.012, −0.005)	<0.001 (−0.016, −0.007)
Third block^3^				
Age (y)	−0.043	−0.046	<0.001 (−0.040, −0.036)	<0.001 (−0.042, −0.039)
Sex	−0.296	−0.303	<0.001 (−6.431, −6.270)	<0.001 (−6.677, −6.491)
Total percent fat (%)	−0.245	−0.243	<0.001 (−0.315, −0.308)	<0.001 (−0.316, −0.307)
Lean mass (kg)	0.482	0.478	<0.001 (0.001, 0.001)	<0.001 (0.001, 0.001)
L-BMD (g/cm^2^)	0.037	0.036	<0.001 (2.529, 2.806)	<0.001 (2.484, 2.803)

Lean mass refers to total lean mass excluding BMC.

Abbreviations: BMC, bone mineral content; CI, confidence interval; L-BMD, lumbar bone mineral density.

1 First block (entire sample): *F*(2,343735) = 250,677.0; *R*^2^ = 0.593; adjusted *R*^2^ = 0.539; SE = 6.848; *P* < 0.001. First block (75% of the sample): *F*(2,258423) = 193,765.2; *R*^2^ = 0.600; adjusted *R*^2^ = 0.600; SE = 6.867; *P* < 0.001.

2 Second block (entire sample): *F*(2,343734) = 83.3; *R*^2^ = 0.000; adjusted *R*^2^ = 0.539; SE = 6.848; *P* < 0.001. Second block (75% of the sample): *F*(2,258422) = 24.8; *R*^2^ = 0.000; adjusted *R*^2^ = 0.600; SE = 6.866; *P* < 0.001.

3 Third block (entire sample): *F*(2,343732) = 71,284.2; *R*^2^ = 0.119; adjusted *R*^2^ = 0.713; SE = 5.758; *P* < 0.001. Third block (75% of the sample): *F*(2,258420) = 53,099.8; *R*^2^ = 0.117; adjusted *R*^2^ = 0.716; SE = 5.781; *P* < 0.001.

### Cross-validation results

The results of the cross-validation showed no significant differences in the adjusted *R*^2^ (<2%) between the full model (entire sample) and the training model (a random 75% of the sample), which ensured that the model did not lose predictability during the cross-validation process (Table 2). Finally, there was no notable difference between the standard error of the residual means of the original model (entire sample; 5.575) and the generated equivalent standard error of estimate for the residuals of the validation sample (the remaining 25% of the sample; 5.715), ensuring the model’s validity.

## Discussion

The findings of this study found that age, sex, total percent fat, lumbar BMD, and total lean mass significantly predicted grip strength in adults without sarcopenia. This study implemented a structured hierarchical analysis model to identify the contribution of each predictor category (demographic factors, adiposity measures, and other body composition factors) and found that demographic factors (age and sex) and body composition factors (namely lumbar BMD and total lean mass) explained 42.3% and 35.3% of the variance in grip strength, respectively. Total percent fat explained only 1.0% of the variance in grip strength. Together, all identified predictors explained 78.6% of the variance in grip strength. The findings of this study supported the initial hypothesis that lumbar BMD and total lean mass will significantly predict grip strength, after controlling for age, sex, and total percent fat.

The association between older age and stronger grip strength in this study is most likely explained by the fact that most of the sample included in this study were young adults (mean age = 28.6 ± 15.6 y) because grip strength increases from childhood onward until it reaches its peak in the middle adulthood (∼30–40 y of age) and then gradually decreases in both men and women [10,11,36]. In addition, women had significantly weaker grip strength in this study. Previous findings indicated that the grip strength of men is greater than that of women and the grip strength of men tends to decrease at a faster rate than that of women, although this decline in grip strength narrows slightly with age [11,37]. To these ends, demographic factors have a great impact on grip strength measures as shown in this study and previous studies. This is important for designing future studies that intend to measure grip strength to consider these factors as potential confounders.

The association between greater percent fat mass and weaker grip strength, after controlling for age and sex in this study, was slight but unneglectable because an increase of 1 kg in grip strength was associated with a decrease of 0.26 in total percent fat. Fat is essential for promoting anabolic effects in muscles specifically at younger ages [38], which could contribute to the association between percent fat and grip strength. However, increased percent fat may lead to fat redistribution between and within muscle cells, especially at older age, which could negatively impact grip strength [19]. The association of grip strength with total percent fat in this study was in line with previous results that found a sex-specific negative association between percent fat and grip strength in adults [39]. In contrast, a population-based United Kingdom study found a cross-sectional association between greater percent fat and stronger grip strength at ages from childhood to the age of 46 y [40]. Younger ages included in the sample of the United Kingdom study simply explain our contrasting findings. Nevertheless, the inconsistency in the direction of association between percent fat and grip strength indicates the complexity of this relationship and the need to cautiously consider how age, sex, obesity level, and specificity of adiposity measurement.

One potential explanation of why lumbar BMD and total lean mass were significant predictors of grip strength is the anabolic responses that promote muscle growth and osteoblast deposition, elicited by mechanical loads [41,42]. These anabolic effects are usually greater in men than those in women owing to higher concentrations of testosterone circulating in the blood, which may explain the observed age-specific and sex-specific association of grip strength with lean mass [43,44]. Estrogen is also a key regulator of bone metabolism and is important for bone health in both men and women, and loss of ovarian estrogen is associated with decreased BMD [45]. Thus, the age-specific and sex-specific association of grip strength with BMD may be driven by the changes in estrogen concentrations. Previous studies found a similar association between stronger grip strength with greater lumbar BMD [26] and lean mass [25]. Although the measured lumbar BMD was not site specific to the bones near the wrist joint in this study, the association was significant. Thus, understanding how grip strength is related to BMD at different sites of the skeleton is important for future studies that aim to prevent osteopenia and osteoporosis, especially for older ages. The novelty of this study is the use of hierarchical analysis, which highlights the weight of the association of each category of confounding variables and the variable of interest with grip strength while excluding sarcopenia. This approach provides strong evidence that maintaining high lumbar BMD is important for good grip strength, which translates into preventing risks associated with weak grip strength.

This study has several strengths. First, the use of DXA in measuring body composition following the standardized protocol by NHANES may minimize risk of measurement error, which is common in observational research. Second, the large sample size and inclusion of approximately equal sexes give sufficient power to detect association.

This study still has some limitations. First, this study did not control for using calcium and vitamin D supplementation, which may confound the results of this study owing to its positive effects on BMD [46]. However, benefits of these supplements need tracking over a long period, which was not performed in NHANES. Future studies should consider using calcium and vitamin D when examining the association between grip strength and BMD. Second, the analysis of this study was retrospective, which may raise concerns about selection bias of samples. However, the NHANES uses a complex, multistage, probability sampling design, which may address this concern. Third, the large sample size may drive the significance of the associations. However, we conducted a cross-validation process to ensure the stability of the model and that the associations were not coincidental. Finally, the BMD measured in this study was not site specific to grip strength. However, the potential biological characteristics are similar, and the lumbar BMD was associated with grip strength in previous research [47].

In summary, this study documented a positive association of grip strength with lumbar BMD and total lean mass, after controlling for age, sex, and total percent fat. This association explained >75% of the variation in grip strength, which helps future research on how to improve grip strength. The results of this study should be considered in the development of public health survival measures and prevention of sarcopenia, given the role of grip strength in sarcopenia.

## Author contributions

The sole author was responsible for all aspects of this manuscript.

## Conflict of interest

The author reports no conflicts of interest.

## Funding

This work was partially supported by the Deanship of Scientific Research at Northern Border University, Arar, Saudi Arabia, through the project number NBU-FFR_2024-2508-03.

## Data availability

Data are available for public in an open access repository (National Health and Nutrition Examination Survey, 2013–2014, at https://wwwn.cdc.gov/nchs/nhanes/).
